# EA-AHS: A Perception-Driven Adaptive Heuristic Framework for Real-Time UAV Path Planning in Complex Urban Environments

**DOI:** 10.3390/s26144355

**Published:** 2026-07-09

**Authors:** Ruijie Song, Haohan Zhang, Xianghua Zeng, Xiaoping Rui

**Affiliations:** 1 School of Earth Sciences and Engineering, Hohai University, Nanjing 210098, China; 2309020214@hhu.edu.cn (R.S.); 2309020132@hhu.edu.cn (X.Z.); 2 College of Information Sciences and Engineering, Hohai University, Nanjing 210098, China; 2304010303@hhu.edu.cn

**Keywords:** UAV path planning, A* algorithm, environmental perception, adaptive heuristic search, dynamic weight adjustment, DDPG

## Abstract

With the progressive opening of urban low-altitude airspace, drone applications increasingly rely on real-time environmental perception for safe navigation in complex, obstacle-dense environments. Traditional planning algorithms struggle to efficiently process spatially heterogeneous sensor data, leading to computational bottlenecks and unsafe trajectories. To bridge raw perception and agile decision-making, we propose an Environment-Aware Adaptive Heuristic Search (EA-AHS) framework that translates local obstacle density into adaptive heuristic weights via a sliding window, while a historical feedback loop enables macro-level parameter adaptation. Experiments demonstrate that EA-AHS reduces planning time by 87.9% in a simple maze and by 81.0% in a complex point-block map compared to standard A*. In a 3D urban scenario, EA-AHS trades a modest computational overhead for substantial safety gains: its cumulative risk is only 48% of that of standard A* and 57% of that of weighted A*, and its minimum obstacle distance increases to 6.04 m. Unlike learning-based methods, EA-AHS requires no pre-training or neural inference, offering a highly practical, lightweight solution for resource-constrained onboard sensor systems.

## 1. Introduction

With the gradual opening of urban low-altitude airspace, drone applications in logistics delivery, infrastructure inspection, disaster emergency response, and smart city management are developing rapidly [1,2]. Compared with traditional airspace, the urban low-altitude environment is characterized by dense obstacle distribution, complex spatial structures, and significant spatial heterogeneity. These features impose stringent demands on the real-time performance and robustness of path planning algorithms. Especially in online mission planning scenarios, path planning must not only ensure the safety and feasibility of the trajectory but also rapidly generate high-quality paths under limited computational resources.

Current mainstream path planning methods mainly include graph search algorithms, intelligent optimization algorithms, and reinforcement learning approaches. Among these, the A* algorithm is widely applied to UAV path planning due to its strong optimality guarantees and high search efficiency [3,4]. This algorithm achieves efficient search by balancing actual cost and heuristic cost. However, in large-scale, complex grid environments, the performance of the standard A* algorithm is highly dependent on the heuristic function and its weight settings. When the environment exhibits significant spatial heterogeneity, fixed heuristic weights struggle to balance search efficiency and path quality. This often leads to a surge in the number of expanded nodes and even search oscillations in obstacle-dense regions.

To address the above issues, researchers have improved the A* algorithm from various perspectives. Examples include reducing computational scale through search space pruning and bidirectional search, accelerating convergence by modifying the form of the heuristic function, and incorporating multi-objective constraints such as energy consumption and risk into the cost function. In addition, algorithms such as Theta* have demonstrated certain advantages in generating shorter, any-angle paths. However, most existing methods focus on optimizing the algorithm structure or the formulation of the heuristic function, lacking explicit modeling and utilization of environmental features themselves. In an urban low-altitude environment with highly uneven spatial distribution, relying solely on a fixed strategy makes it difficult to achieve stable and efficient path planning.

From the perspective of the problem’s essence, urban environments exhibit significant variations in local structure: in open areas, the search process can converge rapidly toward the goal, whereas in obstacle-dense regions, an overly strong heuristic influence may cause the search direction to deviate, thereby reducing overall search efficiency. Therefore, if the search process can perceive local environmental complexity in real time and dynamically adjust heuristic weights accordingly to align the search strategy with environmental characteristics, it is expected to significantly improve path planning performance while maintaining the algorithm’s lightweight nature.

Based on the above analysis, this paper proposes an Environment-Aware Adaptive Heuristic Search (EA-AHS) framework. This framework divides the path planning process into three layers: the environment perception layer, the strategy control layer, and the performance feedback layer. The environment perception layer uses a sliding window to calculate local obstacle density in real time to characterize environmental complexity. The strategy control layer dynamically adjusts heuristic weights based on environmental features. The performance feedback layer utilizes historical planning information to provide a data foundation for macro-level parameter adaptation. Together, these layers establish a two-tier adaptive mechanism that combines micro-level responsiveness with macro-level regulation capabilities.

To validate the effectiveness of the proposed method, systematic comparative experiments were conducted on various 2D maze grid environments and a 3D simulated urban environment. The results show that in the Simple Maze scenario, EA-AHS reduces planning time by 87.9% compared with Standard A*; in the Complex Point-Block Map environment, the planning time is reduced by 81.0%, and the number of expanded nodes is reduced by over 85%. In the 3D urban scenario, fixed-weight methods (Weighted A*) are fast but offer no safety improvement, while EA-AHS trades a modest time overhead (75.01 s) for a substantial reduction in cumulative risk: its RiskSum is only 48% of Standard A* and 57% of Weighted A*, and its minimum obstacle distance increases from 4.62 m to 6.04 m, achieving an excellent balance between efficiency and safety.

The main contributions of this paper are as follows:

1. We propose a dynamic heuristic weight adjustment mechanism based on local obstacle density, enabling environment-adaptive search strategies.
2. We construct a two-layer adaptive framework that integrates environment perception and performance feedback, enhancing the algorithm’s robustness in complex environments.
3. We conduct systematic validation in 2D maze and 3D urban simulation environments, demonstrating that the proposed method achieves a favorable balance between efficiency and path quality.

## 2. Related Work

UAV path planning algorithms are mainly divided into graph search algorithms, intelligent optimization algorithms, and reinforcement learning methods [5,6]. This section reviews research progress closely related to this paper, including heuristic search improvements, adaptive planning methods, and environment-aware path planning.

### 2.1. Heuristic Search Improvements

The A* algorithm is one of the most classic heuristic graph search algorithms. Its search efficiency depends heavily on the design of the heuristic function. To improve search speed, researchers have proposed various improvement strategies. Weighted A* multiplies the heuristic function by a weight factor greater than 1, trading optimality for faster search speed. Hu et al. adopted an exponential weight adjustment to further enhance search efficiency. Bidirectional search mechanisms expand nodes simultaneously from both the start and the goal, effectively reducing the search scale. Chen et al. [7] combined IDA* with bidirectional search to reduce the number of expanded nodes. Liu et al. [8] accelerated the search process by pruning certain grid nodes. Tang et al. linearly combined Manhattan distance and Euclidean distance in the heuristic function and adopted a bidirectional search strategy, which shortened planning time. Most of the above methods set parameters offline for specific scenarios and lack online responsiveness to local environmental changes. Theta [9] further extends A by incorporating line-of-sight checks, enabling any-angle path generation that yields shorter and smoother trajectories, albeit at higher computational cost.

### 2.2. Adaptive Planning Methods

Adaptive planning methods aim to dynamically adjust algorithm parameters based on environmental changes or historical performance. In the field of robot path planning, the D* Lite algorithm copes with environmental changes through incremental replanning [10]. In UAV path planning, some studies have combined the A* algorithm with the artificial potential field method to achieve dynamic obstacle avoidance [11,12]. However, their parameters still require manual tuning. Some research has introduced reinforcement learning ideas (e.g., DDPG) to adjust heuristic weights through online learning. However, these methods usually require large amounts of training data and incur high computational costs. The performance feedback layer proposed in this paper draws on the idea of exploration and exploitation. It uses a lightweight sliding window to record historical planning times and automatically fine-tunes the base weight when environmental changes are detected, thereby avoiding complex learning processes [13,14].

### 2.3. Environment-Aware Path Planning

Environment awareness refers to using local or global environmental information to guide the search process. Zhang et al. [15] incorporated altitude change penalties in urban logistics UAV path planning. Xie et al. [16] integrated factors such as population density and obstacle occlusion to assess flight safety risks. Li et al. [17] introduced noise protection zone constraints in multi-objective planning. The above studies mostly incorporate environmental information into the cost function but do not adjust heuristic weights in real time. Unlike previous studies that passively incorporate environmental information into cost functions, this paper actively utilizes local obstacle density to drive heuristic weight adjustments. Crucially, it establishes a dual-level adaptation mechanism: micro-level responsiveness achieved through real-time density perception, combined with a macro-level parameter regulation mechanism powered by historical feedback, seamlessly bridging the gap between local feature utilization and global strategy optimization.

In summary, existing research has made progress in heuristic search improvements, adaptive planning, and environment awareness. However, local environment perception and historical performance feedback have not yet been organically combined. The EA-AHS framework proposed in this paper fills this gap and provides a new approach for real-time path planning of urban low-altitude UAVs.

## 3. Environment-Aware Adaptive Heuristic Search Framework (EA-AHS)

**Figure 1:**
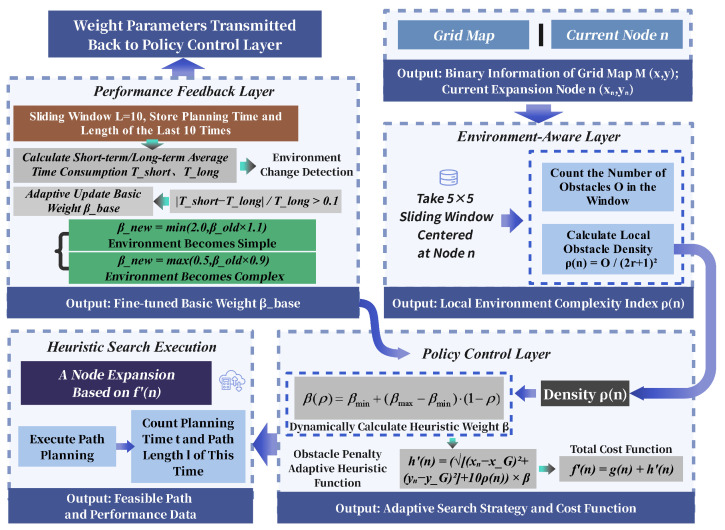
EA-AHS Framework Structure Diagram.

The EA-AHS framework formulates the heuristic search process through three synergistic layers: the environmental perception layer, the policy control layer, and the performance feedback layer, as shown in Figure 1. The environmental perception layer is responsible for quantifying local environmental complexity metrics in real time; the policy control layer dynamically calibrates heuristic weights based on these metrics to steer search behavior; and the performance feedback layer detects macro-level environmental shifts and adaptively updates baseline parameters by logging historical planning performance. Interconnected via data flows, these layers constitute a closed-loop adaptive system. This modular architecture can function independently or be seamlessly integrated with complementary optimization strategies (such as bidirectional search or node pruning).

### 3.1. Environmental Modeling

#### 3.1.1. Grid-Based Environmental Representation

In this paper, the grid method is adopted for discretized environment modeling [18]. For simplicity, the description is based on a 2D grid. Extension to 3D can be achieved by introducing an altitude dimension [19]. The planning area is defined as a rectangular grid of size L×L, where each cell is referred to as a node, and the side length is 1 m (scalable according to actual requirements). Obstacle information is represented by a binary matrix $M\in {\left\{0,1\right\}}^{L\times L}$:

(1) $$M_{ij}=\left\{\begin{array}{cc} 1, & \mathrm{the}\;\mathrm{cell}\;(i,j)\;\mathrm{is}\;\mathrm{occupied}\;\mathrm{by}\;\mathrm{an}\;\mathrm{obstacle},\\ 0, & \mathrm{the}\;\mathrm{cell}\;(i,j)\;\mathrm{is}\;\mathrm{in}\;\mathrm{free}\;\mathrm{space}. \end{array}\right.$$

The UAV can move in eight directions. The movement cost is defined as follows:

(2) $$c\left(n_{i},n_{j}\right)=\left\{\begin{array}{cc} 1, & \mathrm{orthogonal}\;\mathrm{movement}\;(\mathrm{up},\;\mathrm{down},\;\mathrm{left},\;\mathrm{right}),\\ \sqrt{2}, & \mathrm{diagonal}\;\mathrm{movement}. \end{array}\right.$$

A path $P=\{n_{\mathrm{start}},n_{1},n_{2},\ldots ,n_{\mathrm{goal}}\}$ consists of a sequence of adjacent nodes and must satisfy the collision-free constraint, i.e., $\forall n\in P,M_{n}=0$.

#### 3.1.2. Cost Function

For a path *P*, the total cost is defined as the sum of the actual movement costs:

(3) $$g\left(P\right)=\sum_{k=1}^{|P|-1}c\left(n_{k},n_{k+1}\right)$$

where |P| denotes the number of nodes in the path.

During the search process, the A* algorithm uses a heuristic function h(n) to estimate the remaining cost from the current node *n* to the destination $n_{\mathrm{goal}}$. The standard heuristic function uses Euclidean distance:

(4) $$h\left(n\right)={∥n-n_{\mathrm{goal}}∥}_{2}=\sqrt{{\left(x_{n}-x_{\mathrm{goal}}\right)}^{2}+{\left(y_{n}-y_{\mathrm{goal}}\right)}^{2}}$$

where $\left(x_{n},y_{n}\right)$ and $\left(x_{\mathrm{goal}},y_{\mathrm{goal}}\right)$ are the coordinates of node *n* and destination $n_{\mathrm{goal}}$, respectively.

### 3.2. Environment-Aware Model

The core of the environment-aware layer is to compute the local obstacle density near a node. When expanding node *n*, a square window with side length 2r+1 is defined centered at its coordinates $\left(x_{n},y_{n}\right)$. In this paper, r=2 is used, resulting in a window size of 5×5. The number of obstacle cells within the window *O* is counted, and the local obstacle density ρ(n) is calculated:

(5) $$\rho \left(n\right)=\frac{O}{{\left(2r+1\right)}^{2}}$$

This density reflects the level of congestion in the vicinity of a node (ρ(n)∈[0,1]); a higher value indicates a denser concentration of obstacles. This metric can be extended to other environmental features based on application requirements, such as height variance, risk weighting, and population density.

### 3.3. Dynamic Weight Adjustment Strategy

To enable smooth and continuous adaptive regulation of the heuristic weight according to local environmental complexity, we design a linear mapping mechanism. Unlike hard-threshold switching schemes, this mechanism maps the local obstacle density ρ(n) to a dynamic weight β via a linear function, thereby avoiding priority queue jitter and unstable search behavior caused by abrupt weight changes.

For the current expanded node *n*, its dynamic heuristic weight β is given by:

(6) $$\beta \left(\rho \right)=\beta_{\min}+\left(\beta_{\max}-\beta_{\min}\right)\cdot \left(1-\rho \right)$$

where ρ=ρ(n) is the local obstacle density defined in Equation (5), and $\beta_{\min}=0.5$ and $\beta_{\max}=2.0$ are the preset lower and upper bounds. The linear mapping has the following properties:

- When ρ→0 (open area), $\beta \to \beta_{\max}=2.0$, biasing the algorithm toward faster goal convergence using stronger heuristic guidance.
- When ρ→1 (cluttered area), $\beta \to \beta_{\min}=0.5$, reducing reliance on the heuristic and enhancing local exploration to circumvent dense obstacles.
- The weight changes linearly with density, maintaining a constant rate of change without introducing curvature or oscillation, which is simple to implement and incurs minimal computational cost (only one multiply–add operation).

Furthermore, to actively penalize traversals near dense obstacle clusters, an obstacle penalty term 10ρ(n) is integrated into the heuristic function. The resulting composite heuristic, incorporating both the dynamic weight and the penalty term, is expressed as:

(7) $$h^{\prime }\left(n\right)=\left(d\left(n,n_{\mathrm{goal}}\right)+10\rho \left(n\right)\right)\cdot \beta \left(\rho \right)$$

where $d(n,n_{\mathrm{goal}})$ denotes the Euclidean distance from node *n* to the goal.

The final evaluation function for a node remains:

(8) $$f\left(n\right)=g\left(n\right)+h^{\prime }\left(n\right)$$

where g(n) denotes the actual movement cost from the start point to node *n*.

This design only requires computing the local density and obtaining the weight via a linear formula upon each node expansion, incurring only O(1) overhead per expansion. No additional heavy data structures are introduced, thereby preserving the lightweight nature of the framework.

### 3.4. Global Feedback Mechanism

The performance feedback layer enhances the algorithm’s robustness in dynamic environments by recording historical planning performance, detecting macro-level environmental changes, and adaptively adjusting base weights. This mechanism is inspired by the exploration concept in reinforcement learning but is implemented using a lightweight sliding window.

Specifically, a circular queue *Q* with a length of L=10 is created to record the execution time $t_{i}$ and path length $l_{i}$ of the most recent *L* planning runs. After each planning run, the new results are added to the queue; if the queue is full, the oldest record is removed. We define the short-term average time $T_{\mathrm{short}}$ as the mean of the last k=5 planning times, and the long-term average time $T_{\mathrm{long}}$ as the mean of the previous *k* planning times (i.e., the 6th to the 10th entries in the queue). A significant change in the environment is deemed to have occurred when the following condition is satisfied:

(9) $$\frac{|T_{\mathrm{short}}-T_{\mathrm{long}}|}{T_{\mathrm{long}}}>0.1$$

At this point, we adjust the global heuristic weight $\beta_{\mathrm{base}}$ (initially set to 1.0) according to the direction of the change:

(10) $$\beta_{\mathrm{new}}=\left\{\begin{array}{cc} \max(0.5,\beta_{\mathrm{old}}\times 0.9), & \mathrm{if}\;T_{\mathrm{short}}>T_{\mathrm{long}}\\ \; & \;(\mathrm{environment}\;\mathrm{becomes}\;\mathrm{more}\;\mathrm{complex}),\\ \min(2.0,\beta_{\mathrm{old}}\times 1.1), & \mathrm{if}\;T_{\mathrm{short}}<T_{\mathrm{long}}\\ \; & \;(\mathrm{environment}\;\mathrm{becomes}\;\mathrm{simpler}). \end{array}\right.$$

The adjustment magnitude is fixed at 10%, and β is always maintained within the range [0.5,2.0] to ensure algorithm stability. This adjustment is performed only during planning intervals and does not affect the real-time performance of individual searches. The complete EA-AHS algorithm incorporates the aforementioned global feedback mechanism on top of local density adjustment. The above method is described using a 2D grid environment as an example, but its core mechanism can be naturally extended to 3D space.

## 4. Experiments

### 4.1. Simulation Setup

To comprehensively evaluate the performance of the EA-AHS framework, we designed two maze-style grid environments of varying complexity and selected several mainstream and state-of-the-art path planning algorithms for comparison. The hardware platform for experiments was an Intel Core i7-1165G7 @ 2.8 GHz, running Windows 11. All algorithms were implemented in Python 3.12, with core dependencies including NumPy, Matplotlib, and heapq.

#### 4.1.1. Maze Environment Generation

To simulate the coexistence of structured and unstructured obstacles in urban low-altitude environments, this study adopts two types of 200×200 grid maps (grid resolution 1 m), denoted as “Simple Maze” and “Complex Point-Block Map”. All maps are generated via a two-step procedure:

1. Fixed backbone: outer boundary, rectangular obstacle rooms, main corridors, and fixed start/goal areas;
2. Random point-block perturbation: small rectangular obstacle blocks are randomly placed in free cells, with their positions and sizes drawn from predefined distributions (a fixed random seed ensures reproducibility).

This design introduces local variations while keeping the overall difficulty level consistent across runs, thereby better reflecting the spatial heterogeneity of real urban environments.

- Simple Maze: backbone obstacle density ≈18%, with a small number of perturbation blocks (block_count=24), good connectivity and multiple alternative paths.
- Complex Point-Block Map: based on the simple maze backbone; additional ≈42 random point-block obstacles (block_count=42) and a few isolated rooms are introduced, raising the obstacle density to ≈25% and creating more dead ends and non-convex obstacles, which imposes higher demands on the algorithm’s exploration capability.

**Figure 5:**
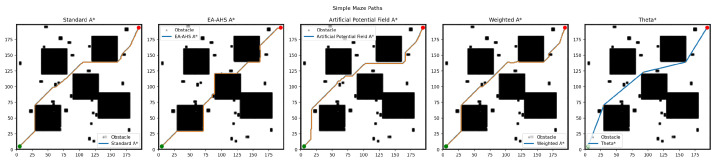
Planned paths in the Simple Maze environment.

**Figure 6:**
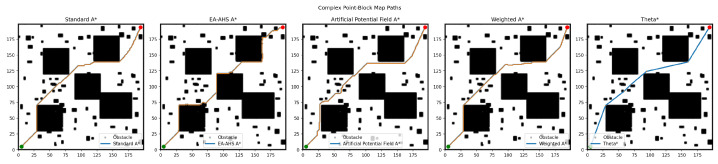
Planned paths in the Complex Point-Block Map environment.

The path shown in Figure 5 and Figure 6 is from one representative run; the evaluation averages over 30 independent runs with different random perturbations, which add local variations not fully captured by a single visualization.

The start and goal points are fixed at (5,5)→(194,194) for the Simple Maze and (10,10)→(189,189) for the Complex Point-Block Map. The backbone of all maps is generated using a fixed seed (np.random.seed(42)) to ensure baseline consistency, while the perturbation blocks in each run use an independent seed, producing different local layouts.

##### Comparison of Algorithms

To thoroughly validate the effectiveness and efficiency of the EA-AHS framework, the following algorithms are selected for horizontal comparison.

- Standard A*: The baseline algorithm, utilizing Euclidean distance as the heuristic function, guaranteeing path optimality.
- EA-AHS A*: The complete framework proposed in this paper, comprising the environment perception layer, policy control layer, and performance feedback layer.
- Weighted A*: A classic heuristic weighting improvement. In this experiment, the heuristic weight is fixed at ω=1.25, which corresponds to the mean value of the EA-AHS heuristic weight range [0.5,2.0], serving as a fixed-weight baseline.
- Artificial Potential Field A*: An environment-aware baseline that maps local obstacle density to heuristic weight adjustments (linear or adaptive), used to compare different environment perception strategies.
- Theta* (2D only): An any-angle path planning algorithm based on line-of-sight reachability. It can generate shorter and smoother paths, albeit with higher computational overhead.

3D experiment note: Because Theta* requires over 300 s per planning run in the 3D environment, it cannot be completed within the 30-run repeated experiment framework. Therefore, Theta* is excluded from the 3D comparison. The 3D comparison includes Standard A*, EA-AHS A*, Weighted A*, and Artificial Potential Field A*.

#### 4.1.2. Parameter Settings

The key parameters of the EA-AHS A* algorithm are summarized in Table 1. The environment perception layer employs a 5×5 sliding window (r=2) to compute the local obstacle density ρ. The policy control layer dynamically adjusts the heuristic weight via a linear mapping function. The performance feedback layer monitors historical planning times using a sliding window of length 10 to adjust the global base weight.

All parameters listed above are kept fixed across all experiments and are not tuned for any specific map. The 30 independent runs (each with different random map perturbations) in both 2D and 3D environments use the same parameter set, demonstrating the generalization capability of the proposed framework. For ablation studies, variants such as “No Penalty” or “No Feedback” are obtained by removing the corresponding modules while keeping all other parameters unchanged.

#### 4.1.3. Evaluation Metrics

To quantitatively assess algorithm performance, the following core metrics are recorded for each experimental run:

- Planning Time (s): The wall-clock CPU time consumed from algorithm initiation to the return of a complete path.
- Peak Memory (MB): The maximum memory usage during planning, recorded using tracemalloc.
- Path Length (m): The total Euclidean distance of the successfully planned path.
- Number of Expanded Nodes: The total number of nodes popped from the open list and expanded during the search, reflecting the computational cost of the search.
- Minimum Obstacle Distance (m): The smallest distance from any sampled point along the path to the nearest obstacle, reflecting the safety margin at the most critical point.
- Average Obstacle Distance (m): The arithmetic mean of distances to the nearest obstacle over all sampled points, characterizing the overall obstacle-avoidance posture.
- Cumulative Risk Value: The sum of distance deficits below the safety threshold $d_{\mathrm{safe}}=8\;m$, defined as $\mathrm{RiskSum}=\sum \max(0,d_{\mathrm{safe}}-d_{\mathrm{obs}})$. A smaller value indicates higher safety.
- Success Rate: The probability of successfully finding a feasible path (all algorithms achieved 100% in this study, thus it is omitted from result tables).

All experiments (both 2D and 3D) are conducted with 30 independent repetitions, each using a different random map seed. (In the 3D environment, due to computational constraints, map seeds are independent while obstacle configurations retain overall similarity.) Results are reported as “mean ± standard deviation”. To confirm statistical significance of the improvements, Mann–Whitney U tests (non-parametric, suitable for non-normally distributed data) are performed on planning time and peak memory, and Cohen’s d effect sizes are reported.

### 4.2. Theoretical Analysis

#### 4.2.1. Bounded Heuristic Inflation and Affine-Relaxed Suboptimality

The standard A* algorithm employs an admissible heuristic function (where $h\left(n\right)\leq h^{*}\left(n\right)$, with $h^{*}\left(n\right)$ being the true optimal cost) and is guaranteed to find the optimal path. After introducing the dynamic weight β(ρ) and the obstacle penalty term λρ(n), the composite heuristic function defined in Equation (7) becomes inflated, thereby violating strict admissibility. Consequently, global optimality cannot be guaranteed.

However, the heuristic inflation in EA-AHS is not arbitrary or unbounded. We can derive an explicit theoretical bound for the proposed heuristic. Since the local obstacle density is bounded by ρ(n)∈[0,1] and the dynamic weight is bounded by $\beta \left(\rho \right)\in \left[\beta_{\min},\beta_{\max}\right]=\left[0.5,2.0\right]$, we can establish an affine relaxation bound for $h^{\prime }\left(n\right)$:

(11) $$\begin{array}{cc} h^{\prime }\left(n\right) & =\left(d\left(n,n_{\mathrm{goal}}\right)+\lambda \rho \left(n\right)\right)\cdot \beta \left(\rho \right)\\ \; & \leq \left(h^{*}\left(n\right)+\lambda \times 1\right)\cdot \beta_{\max}\\ \; & =\beta_{\max}\cdot h^{*}\left(n\right)+\beta_{\max}\cdot \lambda \end{array}$$

where $h^{*}\left(n\right)=d\left(n,n_{\mathrm{goal}}\right)$ represents the standard admissible heuristic. Substituting our parameters ($\beta_{\max}=2.0$, λ=10), this bound simplifies to:

(12) $$h^{\prime }\left(n\right)\leq 2.0\cdot h^{*}\left(n\right)+20$$

This theoretical bound guarantees that the heuristic estimate deviates from the optimal heuristic by at most a linear multiplicative factor and a constant additive term. This property categorizes EA-AHS as an affine-relaxed suboptimal heuristic search. Unlike unbounded greedy methods, EA-AHS’s search behavior is strictly constrained by Equation (12), which prevents unbounded deviation from the optimal path and ensures algorithmic stability.

Regarding the actual path cost, the additive term (20) in the affine bound prevents a simple ϵ-suboptimal guarantee (where path cost $\leq \epsilon \cdot C^{*}$) as in classical Weighted A*. Therefore, the path length increase (e.g., typically observed within 9.5∼12.8% in 2D and 4.1% in 3D environments) is an empirical observation rather than a formal worst-case guarantee. In theory, the worst-case path length may exceed the optimum by a factor related to the cumulative effect of the affine bound. Future work will further investigate tight bounded-suboptimal guarantees under this dynamic affine relaxation model.

#### 4.2.2. Coupled Effect of Dynamic Weight and Obstacle Penalty

In EA-AHS, the heuristic function incorporates both a dynamic weight factor β(ρ) and an additive penalty term 10ρ(n), which jointly affect the estimated remaining cost in a multiplicative coupling manner, as defined in Equation (7). At first glance, the two mechanisms exhibit opposite trends in dense regions (ρ→1): $\beta \left(\rho \right)\to \beta_{\min}=0.5$ reduces the heuristic value, while 10ρ increases it. However, this opposition actually provides a complementary balance:

- In open areas (ρ≈0): β≈2.0 and the penalty term is negligible, yielding $h^{\prime }\left(n\right)\approx 2.0\cdot d$, which emphasizes goal-directed behavior and accelerates convergence.
- In moderately dense obstacle regions: the penalty 10ρ significantly raises the heuristic value while β decreases to around 1.0, giving $h^{\prime }\left(n\right)\approx d+10\rho$. This slightly inflated heuristic guides the search to detour rather than penetrate dense areas.
- In extremely dense regions (ρ→1): β drops to 0.5 and the penalty reaches 10. Then $h^{\prime }\left(n\right)\approx 0.5\cdot \left(d+10\right)$. Even when the goal is far away, the heuristic value is suppressed, preventing excessive exploration inside dead ends.

By analyzing extreme cases, we obtain the ratio of the heuristic value to the standard Euclidean distance:

(13) $$R\left(\rho \right)=\frac{h^{\prime }\left(n\right)}{d(n,n_{\mathrm{goal}})}=\beta \left(\rho \right)\left(1+\frac{10\rho }{d}\right)$$

When *d* is large, the penalty term is relatively small, and the weight β dominates. When *d* is small (i.e., near obstacles), the penalty term dominates, effectively discouraging the search from entering hazardous regions. Thus, the two mechanisms are not conflicting but achieve an environment-adaptive heuristic modulation via multiplicative coupling: speeding up in safe areas while cautiously circumventing obstacles in dense regions.

#### 4.2.3. Rationale of Linear Mapping

In the EA-AHS framework, the mapping between the heuristic weight β and the local obstacle density ρ is central to the adaptation mechanism. Although quadratic or cubic functions can also provide continuous mappings, we ultimately adopt the linear mapping (Equation (6)) for three main reasons: computational lightness, constant rate of change, and experimentally verified performance advantage. To fairly compare different mapping functions, we tested linear (1−ρ), quadratic ${\left(1-\rho \right)}^{2}$, and cubic ${\left(1-\rho \right)}^{3}$ mappings under identical conditions. The experiments were conducted in both the Simple Maze and the Complex Point-Block Map environments, with 30 independent runs per mapping (each using a different random layout). The results are summarized in Table 2.

As shown in Table 2, the linear mapping achieved the shortest average planning time (Simple Maze: 0.1636 s; Complex Point-Block Map: 0.2024 s) and the lowest memory usage (Simple Maze: 0.5926 MB; Complex Point-Block Map: 0.7325 MB) in both environments. In terms of safety metrics such as path length and minimum distance, all three mappings performed similarly; however, the linear mapping consumed significantly less energy than the quadratic and cubic ones. Mann–Whitney U tests confirmed that the improvements in linear mapping over cubic mapping in time, memory, and expanded nodes are statistically significant (p<0.01, with medium to large effect sizes). Therefore, the linear mapping is selected for the EA-AHS framework because of its minimal computational cost, constant and smooth regulation characteristic, and empirically superior performance.

#### 4.2.4. Complexity Analysis

The time complexity of the standard A* algorithm is O(ElogV), where *E* is the number of edges and *V* is the number of nodes. The additional computational overhead introduced by the proposed framework consists of the following components. First, the local density calculation: for each expanded node, the number of obstacles within the window is counted. Since the window size is a constant ${\left(2r+1\right)}^{2}$, this operation is O(1). Second, the performance feedback layer: after each planning episode, the sliding window is updated and a decision is made on whether to adjust the base weight. This involves O(L) operations, where *L* is a constant. Therefore, the overall time complexity remains O(ElogV). The space complexity increases only by the sliding window length *L*, which is a constant and does not affect the asymptotic complexity.

#### 4.2.5. Parameter Robustness

**Figure 2:**
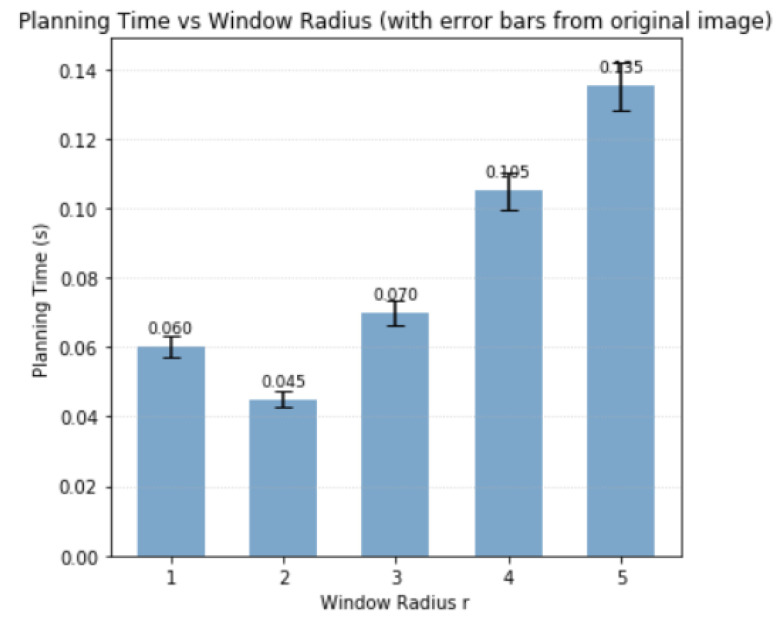
Effect of window radius *r* on planning time.

**Figure 3:**
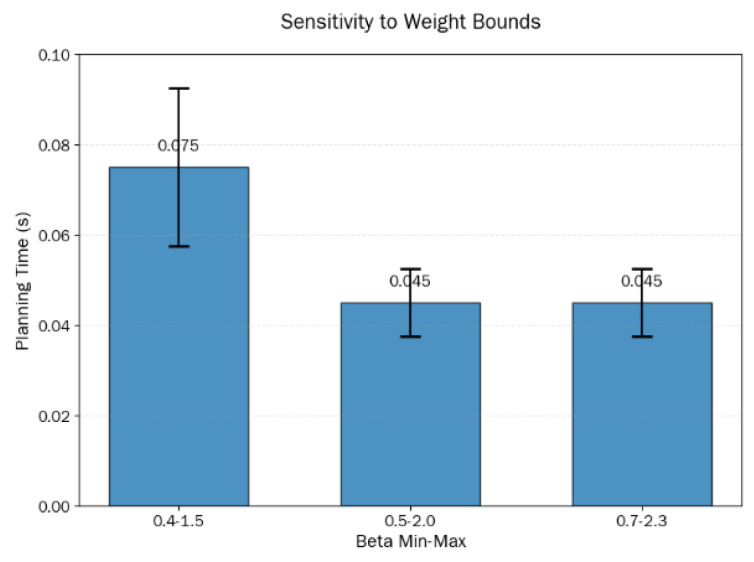
Effect of weight bounds on planning time.

**Figure 4:**
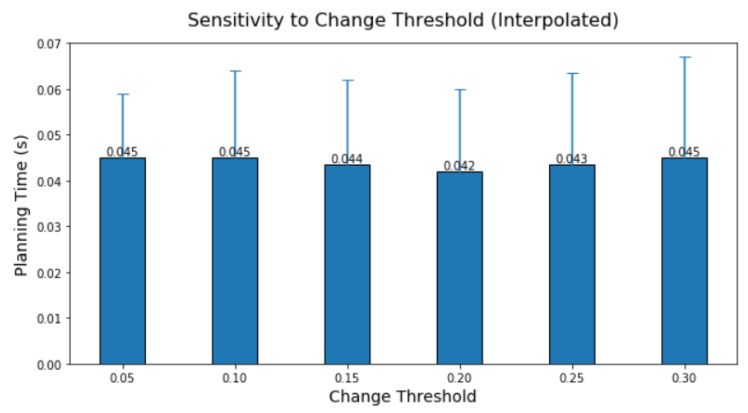
Effect of change detection threshold δ on planning time.

To evaluate the sensitivity of the EA-AHS framework to its core parameters, we conducted sensitivity tests on the window radius *r*, the weight adjustment range $\left[\beta_{\min},\beta_{\max}\right]$, and the change detection threshold δ. The experiments used a fixed set of maps (including simple, complex, and dense environments). In each test, only the target parameter was varied while all other parameters were kept at their default values (r=2, β∈[0.5,2.0], δ=0.1). The average planning time of EA-AHS A* was recorded. Results are presented in Figure 2, Figure 3 and Figure 4.

Figure 2 illustrates the effect of the window radius *r* on planning time as *r* varies from 1 to 5. The lowest average time (0.045 s) is achieved at r=2. When r=1, the time increases to 0.060 s, indicating that a window that is too small cannot adequately perceive the local environment. When r≥3, the time increases significantly (reaching 0.135 s at r=5). This is because the computational cost of density calculation rises and the information within the window becomes saturated. The results suggest that r=2 is the optimal choice and that the algorithm performs stably near this value.

Figure 3 compares the planning time under three different weight bounds. Both the default bounds [0.5,2.0] and the bounds [0.7,2.3] yield the best time of 0.045 s. In contrast, the narrower bounds [0.4,1.5] cause the time to rise to 0.075 s. This indicates that an excessively small upper bound limits the guiding effect of the heuristic, while an overly small lower bound makes the search too conservative. The default bounds [0.5,2.0] achieve a good balance between efficiency and path quality.

Figure 4 shows the results for the change detection threshold δ ranging from 0.05 to 0.30. The average time remains stable at approximately 0.045 s across all tested thresholds, with no notable fluctuations. This demonstrates that the performance feedback layer is insensitive to the choice of δ. The default value δ=0.1 can effectively detect environmental changes without causing overly frequent adjustments.

In summary, the main parameters of the EA-AHS framework exhibit low sensitivity within reasonable ranges. The algorithm can operate stably in different environments without requiring fine parameter tuning, which facilitates practical deployment. It should be emphasized that the above conclusions are based on experimental observations, not theoretical guarantees of insensitivity; the behavior of parameters in extreme environments still requires case-specific analysis or additional experiments.

### 4.3. Performance Comparison with Mainstream Algorithms

This section systematically compares Standard A*, EA-AHS A*, Artificial Potential Field A* (APF A*), Weighted A* (ω=1.25), and Theta* in the Simple Maze and Complex Point-Block Map environments. Each environment is run 30 independent times, each with a different random map perturbation (see Section 4.1.1), to evaluate generalization across varied layouts. Metrics including planning time, peak memory, path length, and number of expanded nodes are recorded and reported as mean ± standard deviation in Table 3. All algorithms achieved a 100% success rate.

As shown in Table 3, EA-AHS A* achieves the shortest planning time (Simple Maze: 0.1693 s; Complex Map: 0.2466 s) and the lowest memory usage (Simple Maze: 0.551 MB; Complex Map: 0.747 MB) in both environments, reducing time by 87.9% and 81.0% compared to Standard A*, respectively. The number of expanded nodes is also drastically reduced (by 90.8% and 85.6%). Although EA-AHS produces slightly longer paths (+9.5% in Simple Maze, +12.8% in Complex Map), this trade-off is acceptable for real-time missions.

Weighted A* performs well in the Simple Maze (0.3486 s) but remains slower than EA-AHS in the Complex Map. APF A*, as an environment-aware baseline, shows higher time and memory than Weighted A* without providing significant safety benefits. Theta* yields the shortest paths (286.38 m and 287.60 m) but incurs the highest computational cost (4.37 s and 3.46 s), making it unsuitable for time-sensitive tasks [20].

Figure 5 and Figure 6 visualize the paths generated by the algorithms in the two environments. EA-AHS A* produces a smooth, obstacle-avoiding trajectory in the Simple Maze and adopts a more conservative detour in the Complex Point-Block Map, avoiding extensive ineffective exploration that burdens Standard A*. This validates the effectiveness of its environment-adaptive search strategy.

In summary, across both environments, EA-AHS A* consistently leads in planning efficiency, with particularly outstanding gains in the Simple Maze, while keeping path length increases within a manageable range. Weighted A* offers a robust lightweight alternative, and Theta* maintains superior path quality at the expense of higher computational overhead.

### 4.4. Case Study in a 3D Simulated Urban Environment

To further validate the effectiveness of the EA-AHS framework in realistic three-dimensional environments, this section constructs a large-scale 3D simulated urban scene. The environment spans 1.5km×1.5km horizontally with a vertical ceiling of 500m, discretized at a resolution of 5m×5m×5m. The scene incorporates 58 buildings, 250 trees, 12 intruder UAVs, and 25 random aerial obstacles [17]. A simplified wind-drag energy model is also included. The start and goal positions are (500,500,30) and (1500,1500,270), respectively, ensuring the path traverses zones of varying obstacle density.

#### 4.4.1. Safety Evaluation Metrics

To quantify path safety, three metrics are introduced:

- Minimum Obstacle Distance (MinDist): the smallest distance from any sampled point to the nearest obstacle.
- Average Obstacle Distance (AvgDist): the mean distance to the nearest obstacle over all sampled points.
- Cumulative Risk Value (RiskSum): the sum of distance deficits below a safety threshold $d_{\mathrm{safe}}=8.0\;m$, i.e., $\mathrm{RiskSum}=\sum \max(0,d_{\mathrm{safe}}-d_{\mathrm{obs}})$. A smaller value indicates higher safety.

#### 4.4.2. Experimental Results and Analysis

**Figure 7:**
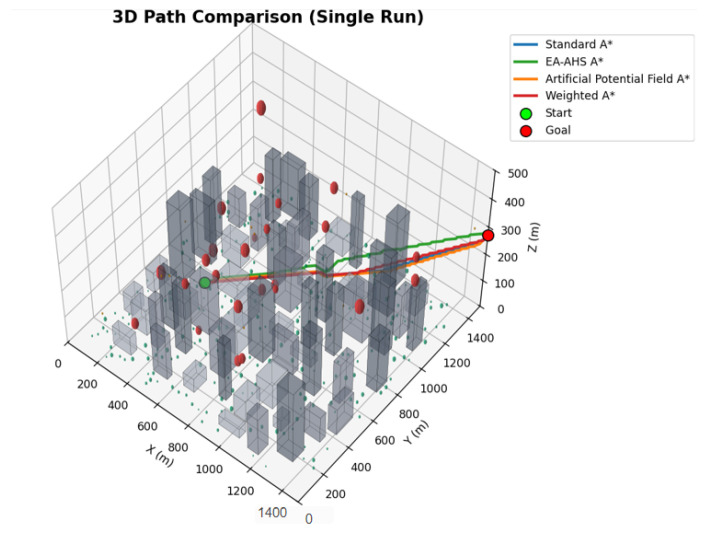
Representative planned paths in the 3D urban environment.

Following the same protocol as the 2D experiments, 30 independent runs are conducted (each with a different random map seed). The compared algorithms include Standard A*, EA-AHS A* (with linear mapping), Weighted A* (ω=1.25), and Artificial Potential Field A* (APF A*). Theta* is excluded because its single-run planning time exceeds 300 s, making it infeasible for 30 repetitions. The results are summarized in Table 4, and a representative path visualization is shown in Figure 7.

As shown in Table 4, EA-AHS A* requires a slightly longer planning time (75.01 s) than Standard A* (58.07 s), but it achieves a substantially lower cumulative risk: the RiskSum of EA-AHS is 38.27, compared to 79.63 for Standard A* and 67.27 for Weighted A*. This means EA-AHS reduces the risk to about 48% of Standard A* and 57% of Weighted A*. Moreover, EA-AHS attains a larger minimum obstacle distance (6.04 m vs. 4.62 m for Standard A* and Weighted A*), indicating that it actively avoids dense obstacle clusters. Although EA-AHS incurs a moderate time overhead, this trade-off is well justified for safety-critical missions. APF A* shows the highest time and memory consumption with only moderate safety improvement. Weighted A* is the fastest but offers no safety gain over Standard A*.

In summary, the EA-AHS framework, through its linear adaptive weight and obstacle penalty, successfully trades a modest increase in planning time for a significant improvement in path safety, providing unique engineering value for low-altitude urban UAV operations. Future work will focus on further reducing the time gap while maintaining the safety advantage.

### 4.5. Ablation Study: Roles of Penalty Term and Feedback Layer

To quantitatively evaluate the individual contributions of the obstacle penalty term (10ρ) and the performance feedback layer, we design three variants for ablation: (1) Full EA-AHS (with both penalty and feedback); (2) No Penalty (remove the obstacle penalty, keep feedback); (3) No Feedback (remove the historical feedback adjustment, keep only the local-density-driven linear weight and penalty). All variants are run on the same 3D urban environment (Section 4.4) for 30 independent repetitions using the same random seeds and the linear mapping. Results are summarized in Table 5.

From Table 5, we observe:

- Effect of the penalty term: Comparing Full EA-AHS with No Penalty, removing the penalty increases planning time from 75.01 s to 89.01 s (+18.7%), memory from 2.83 MB to 3.37 MB, path length from 1579.47 m to 1690.77 m (+7.0%), and RiskSum from 38.27 to 54.55 (+42.5%), while MinDist decreases from 6.04 m to 5.02 m. This demonstrates that the penalty term effectively guides the search away from dense obstacles, significantly improving safety while also modestly reducing planning time and path length.
- Effect of the feedback layer: Comparing Full EA-AHS with No Feedback, removing the feedback layer drastically increases planning time to 291.32 s (about 3.9 times that of the full version), memory to 9.63 MB (3.4 times), and expanded nodes to 1093 (3.4 times). Path length slightly decreases (1647.55 m vs. 1579.47 m), and RiskSum drops to an extremely low value (1.25). However, this “safety” comes at an unacceptable computational cost: without feedback, the algorithm becomes overly conservative, performing massive useless exploration in dense regions. The feedback layer, through macro-level historical adjustment, effectively prevents such over-conservatism, improving computational efficiency by an order of magnitude while maintaining high safety.

In summary, the penalty term and the feedback layer play complementary roles: the penalty term operates at the micro level to steer the search away from obstacles, enhancing safety; the feedback layer works at the macro level to adjust the global weight, preventing over-conservatism and ensuring real-time performance. Their synergy enables EA-AHS to achieve an excellent balance between safety and efficiency.

### 4.6. Lightweight Comparison with DDPG-A*

**Figure 8:**
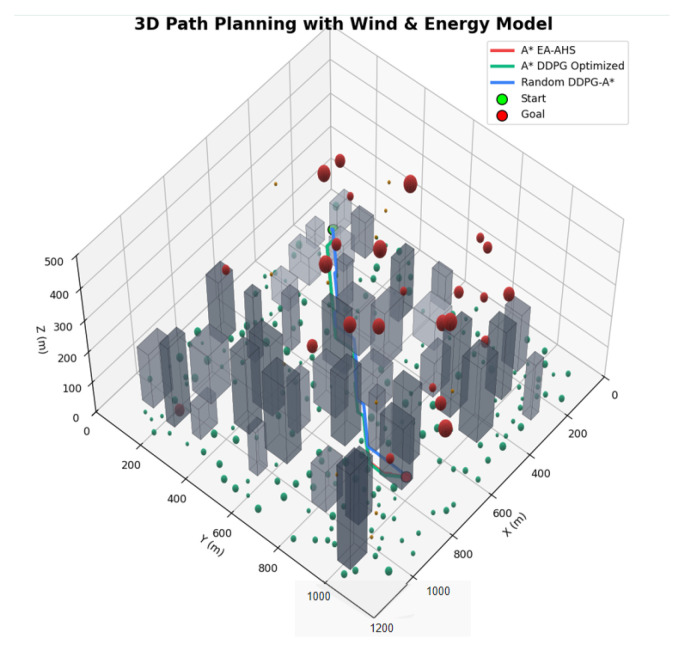
Comparison of planned paths between EA-AHS and DDPG-A* in the reduced-scale 3D urban environment.

Beyond traditional heuristic search improvements, recent studies have explored using deep reinforcement learning to online adjust the heuristic weights of A* for adaptive planning. A representative method is DDPG-A*, which treats the heuristic weight and safety penalty coefficient as continuous actions and learns optimal parameter combinations through trial and error. To verify the lightweight advantage of EA-AHS, we conduct a systematic comparison in a 3D urban environment (same as Section 4.4 but with map size reduced to 1.2km×1.2km to accelerate DDPG training). To further validate the robustness, we perform 10 independent training+testing runs(each with 10 pre-training episodes) and include Random DDPG-A* (no pre-training, randomly initialized policy) as a control. Results are summarized in Table 6, and a representative path visualization is shown in Figure 8.

From Table 6, we make the following observations:

1. Planning efficiency: EA-AHS achieves an average planning time of only 10.18 s, which is 42% faster than DDPG-A* (17.56 s) and an order of magnitude faster than Random DDPG-A* (150.26 s). The number of expanded nodes for EA-AHS (230) is far lower than that of DDPG-A* (452) and Random DDPG-A* (3452). This advantage stems from EA-AHS’s purely analytical local density computation (O(1) complexity) and lightweight sliding window, avoiding the heavy overhead of neural network forward/backward propagation, gradient calculation, and experience replay.
2. Training overhead: DDPG-A* requires an average of 179.22 s of pre-training (10 episodes), with a total time (training+search) of 196.77 s, while EA-AHS needs no training at all. Even when only considering search time, EA-AHS remains significantly faster than DDPG-A*. Random DDPG-A*, despite having no training time, exhibits extremely long search times (150.26 s) with huge variance, indicating unstable performance.
3. Path quality: DDPG-A* yields path length (1345.20 m) and energy (814.80) almost identical to those of EA-AHS (1339.87 m, 809.40), with differences below 0.4%. Random DDPG-A* also produces similar path quality. This indicates that although deep reinforcement learning can discover slightly better parameter combinations through offline training, the gain is negligible (<0.5%) at the cost of nearly 20 times longer planning time.
4. Lightweight deployment: EA-AHS does not rely on GPU acceleration, requires no experience replay buffer, and involves no tedious tuning of hyperparameters (e.g., learning rate, discount factor, soft update coefficient). Its entire adaptive logic consists merely of obstacle counting within a window and a few arithmetic operations, enabling real-time execution on microcontrollers or low-power embedded platforms. In contrast, DDPG-A* must maintain two deep networks (Actor and Critic, each with approximately $2\times 10^{4}$ trainable parameters) and depends on CUDA acceleration to achieve acceptable training speeds. This severely limits its applicability on resource-constrained edge computing devices.

This comparison demonstrates that although DDPG-A* can find slightly better paths via deep reinforcement learning, its heavy computational burden and training latency render it impractical for online real-time planning of urban low-altitude UAVs. In contrast, EA-AHS, by virtue of fully analytical adaptive rules based on environmental features, reduces planning time by over an order of magnitude while keeping path quality degradation below 0.3%. It requires no pre-training or neural network inference. These results strongly validate the core design philosophy of the EA-AHS framework: lightweight adaptation. By explicitly modeling local obstacle density and employing simple linear mappings, effective heuristic regulation is achieved without the excessive complexity of “black-box” learning methods. This makes EA-AHS highly suitable for real-time embedded systems.

## 5. Discussion

The EA-AHS framework demonstrates comprehensive advantages across 2D and 3D environments. In the Simple Maze, planning time drops by 87.9% and node expansions by 90.8% versus Standard A*; in the Complex Point-Block Map, time improves by 81.0% with 85.6% fewer node expansions. The 3D urban case highlights its safety–efficiency balance: although EA-AHS (75.01 s) is slower than Standard A* (58.07 s) and Weighted A* (36.87 s), its cumulative risk (38.27) is only 48% of Standard A* (79.63) and 57% of Weighted A* (67.27), and its minimum obstacle clearance (6.04 m) is significantly higher than theirs (4.62 m). These results validate the framework’s ability to balance speed and safety across diverse scenarios.

This performance stems from a two-tier adaptive mechanism. Microscopically, local obstacle density computed in real time drives a linear weight function that raises the heuristic weight in open areas for faster convergence and lowers it in cluttered regions to enhance exploration. Macroscopically, a sliding window of historical performance provides a mechanism for detecting environmental shifts, establishing a foundation for macro-level parameter regulation. Furthermore, the integrated obstacle penalty term proactively shapes the search trajectory away from dense clusters, directly translating to reduced risk values in complex scenarios.

These efficiency gains entail moderate path length increases: 9.5% in the Simple Maze, 12.8% in the Complex Point-Block Map, and 4.1% in the 3D scenario (1579.47 m vs. 1517.43 m). This trade-off is highly acceptable for time-critical missions, where a safe, feasible path delivered rapidly outweighs a delayed optimal one. Theta* yields shorter paths but at prohibitive computational cost; Weighted A* is fast yet offers no safety benefit. EA-AHS bridges this gap, trading a minor time penalty for substantial safety improvements.

The comparison with DDPG-A* further highlights the paradigm-shifting practicality of EA-AHS. Although deep reinforcement learning can discover marginally better parameter combinations (yielding a <0.3% improvement in path quality), it comes at the cost of nearly twenty times the computational overhead and significant training latency. DDPG-A* relies on dual deep networks, replay buffers, and GPU acceleration, creating a prohibitive barrier for real-time missions. In stark contrast, EA-AHS relies solely on O(1) window-based obstacle counting and a linear weight evaluation. This purely analytical design enables deterministic execution on resource-constrained embedded platforms, transforming the framework from an academic concept into a deployable engineering solution.

Limitations and future work: First, key parameters (e.g., penalty coefficients) are currently set empirically and may require recalibration for drastically different extreme environments. Second, the current framework is designed for single-query planning in static environments; integrated dynamic replanning capabilities are not yet incorporated. Future work will explore combining EA-AHS with incremental replanning algorithms such as D* Lite, enabling real-time path replanning in environments with moving obstacles through density-guided heuristic modulation. Third, the 3D evaluation currently relies on a single simulated urban layout; future studies will test generalization across varied city morphologies (e.g., low-density suburbs, high-density CBDs). Furthermore, the framework will be extended to multi-objective optimization (balancing energy, communication constraints, and regulatory compliance) and validated through extensive real-world flight tests in authentic low-altitude urban settings.

## 6. Conclusions

This paper proposed an Environment-Aware Adaptive Heuristic Search (EA-AHS) framework to address the computational bottlenecks and safety challenges of path planning in complex urban low-altitude environments. By structuring the planning process into three synergistic layers and employing a micro-macro dual-level adaptation mechanism, the framework dynamically adjusts heuristic weights based on real-time local obstacle density while leveraging a sliding window of historical performance to detect macroscopic environmental shifts. This analytical design achieves lightweight, environment-aware adaptation without incurring the computational burdens typical of learning-based methods.

Experimental validations across 2D mazes and a 3D simulated urban scenario demonstrate the framework’s exceptional balance between efficiency and safety. In 2D environments, EA-AHS reduces planning time by 87.9% in the Simple Maze and 81.0% in the Complex Point-Block Map compared to Standard A*, while reducing expanded nodes by over 90%. In the 3D urban scenario, EA-AHS trades a modest time overhead (75.01 s vs. 58.07 s for Standard A*) for a substantial reduction in cumulative risk: its RiskSum (38.27) is only 48% of Standard A* (79.63) and 57% of Weighted A* (67.27), and its minimum obstacle distance increases to 6.04 m. All improvements are confirmed by Mann–Whitney U tests (p<0.01) with medium-to-large effect sizes.

Most notably, benchmarking against the DDPG-A* reinforcement learning approach highlights the EA-AHS framework’s engineering supremacy: it achieves over an order of magnitude faster planning (with path quality degradation strictly controlled below 0.3%), entirely eliminating the need for pre-training, neural network inference, and GPU acceleration.

By decisively bridging the gap between theoretical optimality and real-time computational constraints, the EA-AHS framework transforms environment-aware path planning into a highly practical solution. Its deterministic and lightweight characteristics make it exceptionally suitable for direct deployment on resource-constrained embedded systems, providing a robust foundational algorithm for the upcoming era of large-scale low-altitude urban UAV applications.

## Figures and Tables

**Table 1:** Parameter Settings of EA-AHS A* Algorithm.

Module Layer	Parameter	Value/Range
Environment Perception Layer	Window radius *r*	2 (5×5 window)
Policy Control Layer	Heuristic weight range β	[0.5,2.0]
	Obstacle penalty coefficient λ	10
Performance Feedback Layer	Sliding window length *L*	10
	Change detection threshold δ	0.1
	Base weight adjustment magnitude	±10%

**Table 2:** Comparison of different mapping functions in 2D environments (mean ± std, 30 independent runs).

Environment	Metric	Cubic ${\left(1-\rho \right)}^{3}$	Quadratic ${\left(1-\rho \right)}^{2}$	Linear (1−ρ)
Simple Maze	Time (s)	0.2288±0.0463	0.1910±0.0291	0.1636±0.0377
	Mem (MB)	0.8768±0.0898	0.7678±0.0764	0.5926±0.1296
	Nodes	2095.3±320.4	1816.4±232.4	1545.5±401.2
	PathLen	331.84±7.24	330.09±9.94	332.82±15.60
	MinDist	1.00±0.00	1.00±0.00	1.00±0.00
	AvgDist	3.55±0.66	3.22±0.41	3.30±0.43
	Energy	75.95±15.36	63.09±10.00	54.92±15.29
Complex Point-Block Map	Time (s)	0.3674±0.1698	0.3020±0.2004	0.2024±0.0700
	Mem (MB)	1.2080±0.3950	1.0297±0.4333	0.7325±0.1889
	Nodes	3260.7±1312.9	2798.7±1823.3	1931.6±733.4
	PathLen	343.29±14.43	344.70±19.55	341.42±15.34
	MinDist	1.00±0.00	1.00±0.00	1.00±0.00
	AvgDist	2.87±0.54	2.44±0.45	2.56±0.54
	Energy	127.58±63.02	103.89±67.62	69.89±27.48

Statistical significance (Mann–Whitney U vs. Cubic): For Linear mapping, p<0.01 for Time, Mem, Nodes in both environments; effect sizes (Cohen’s *d*) range from 0.6 to 0.8 (medium to large). Quadratic mapping also shows significant improvements (p<0.05) but with smaller effect sizes.

**Table 3:** Performance comparison in two 2D environments (30 independent runs, mean ± std).

Environment	Algorithm	Time (s)	Mem (MB)	Path Length (m)	Nodes Expanded
Simple Maze	Standard A*	1.3979±0.0397	2.257±0.049	303.68±0.30	14924.1±181.8
	EA-AHS A*	0.1693±0.0238	0.551±0.103	332.43±8.29	1377.3±173.4
	APF A*	1.7476±0.0444	2.901±0.051	303.68±0.21	28216.3±327.8
	Weighted A*	0.3486±0.1026	0.812±0.146	304.36±0.38	3580.0±995.6
	Theta*	4.3738±0.2847	1.084±0.012	286.38±0.67	10709.1±242.3
Complex Point-Block Map	Standard A*	1.2991±0.0437	2.084±0.067	304.82±2.26	14414.5±438.7
	EA-AHS A*	0.2466±0.1025	0.747±0.231	343.95±16.81	2075.4±923.4
	APF A*	1.5977±0.0678	2.621±0.092	304.97±2.31	26281.5±753.8
	Weighted A*	0.2817±0.1043	0.718±0.223	307.31±6.27	3045.2±1153.2
	Theta*	3.4634±0.1596	1.078±0.021	287.60±2.02	10566.6±418.8

Note: All methods succeeded in both environments. Mann–Whitney U tests for EA-AHS vs. Standard A* yield p<0.001 for time and memory in both environments, with Cohen’s *d* effect sizes exceeding 13 (time) and 7 (memory).

**Table 4:** Performance comparison in the 3D urban environment (30 independent runs, mean ± std).

Metric	Standard A*	EA-AHS A*	APF A*	Weighted A*
Time (s)	58.07±38.84	75.01±2.61	125.14±100.37	36.87±2.14
Mem (MB)	2.64±1.55	2.83±0.6	4.83±3.78	1.77±0.10
Path Len (m)	1517.43±68.56	1579.47±78.36	1532.56±77.34	1517.83±73.48
Nodes	377.2±278.9	323.0±47.7	584.4±558.2	216.8±17.1
MinDist (m)	4.62±1.75	6.04±1.14	5.55±1.56	4.62±1.75
AvgDist (m)	54.44±8.54	50.03±7.58	55.14±8.83	57.76±5.77
RiskSum	79.63±52.65	38.27±17.08	42.18±42.61	67.27±39.76

Note: All algorithms achieved 100% success rate. EA-AHS A* uses linear mapping; data from the full variant in ablation study.

**Table 5:** Ablation results in the 3D urban environment (30 independent runs, mean ± std).

Metric	Full EA-AHS	No Penalty	No Feedback
Time (s)	75.01±2.61	89.01±2.19	291.32±6.72
Mem (MB)	2.83±0.06	3.37±0.04	9.63±0.06
PathLen (m)	1579.47±78.36	1690.77±86.31	1647.55±84.47
Nodes Expanded	323.0±47.7	325.0±35.6	1093.0±108.66
MinDist (m)	6.04±1.14	5.02±1.58	7.61±0.79
AvgDist (m)	50.03±7.58	34.29±10.07	37.64±9.42
RiskSum	38.27±17.08	54.55±18.01	1.25±10.73

Note: All variants achieved 100% success rate. Full EA-AHS uses the linear mapping and includes both penalty and feedback.

**Table 6:** Performance comparison between EA-AHS and DDPG-A* in the 3D environment (10 independent runs, mean ± std).

Metric	EA-AHS A*	DDPG-A*	Random DDPG-A*
SuccRate	100%	60%	100%
Search Time (s)	10.18±2.26	17.56±10.84	150.26±258.33
Train Time (s)	0.00±0.00	179.22±111.93	0.00±0.00
Total Time (s)	10.18±2.26	196.77±122.77	150.26±258.33
Nodes Expanded	230.0±0.0	452.0±278.8	3452.2±5850.9
Path Length (m)	1339.87±0.00	1345.20±0.60	1333.87±19.66
Energy	809.40±0.00	814.80±3.96	786.47±51.08

Note: EA-AHS uses linear mapping and requires no pre-training. DDPG-A* training time includes 10 episodes of online learning. Random DDPG-A* uses a randomly initialized actor network without pre-training. Memory metrics are omitted because DDPG relies on GPU acceleration, making direct comparison with CPU-based EA-AHS unfair.

## Data Availability

The simulation data generated during this study are available from the corresponding author upon reasonable request.

## Abbreviations

The following abbreviations and terms are used in this manuscript:

DDPG	Deep Deterministic Policy Gradient
EA-AHS	Environment-Aware Adaptive Heuristic Search
UAV	Unmanned Aerial Vehicle
MinDist	Minimum Obstacle Distance
AvgDist	Average Obstacle Distance
RiskSum	Cumulative Risk Sum

## References

[1] Du M. (2026). Research on the Development Path of Civil Aviation Safety Supervision in China. J. Nanjing Univ. Aeronaut. Astronaut..

[2] Zhang Q., Xu W., Zhang H., Zou Y., Chen Y. (2020). Path Planning for Complex Low-Altitude Logistics UAVs. J. Beijing Univ. Aeronaut. Astronaut..

[3] Zhao Y., Zhou W., Wang X., Chen T. (2026). Robot path planning based on improved A-star algorithm. Mod. Electron. Technol..

[4] Tang J., Peng Z., Li M., Liu Z., Xie C. (2023). Research on UAV Path Planning Based on an Improved A* Algorithm. Electron. Meas. Technol..

[5] Xie F. (2023). An Agricultural UAV Path Planning System Based on the Internet of Things and Artificial Intelligence. Res. Agric. Mech..

[6] Liu Q., Shu L., Liu G., Li A. (2023). A Review of Path Planning Algorithms for Low-Altitude UAVs. Adv. Aeronaut. Eng..

[7] Chen X., Ai Y., Liang H. (2019). Research on 3D trajectory planning of UAV based on improved ant colony algorithm. Tactical Missile Technol..

[8] Liu Y., Xu D., Cheng G., Cheng G. (2020). A Rapid Trajectory Planning Method for UAVs Based on an Improved A* Algorithm. Flight Dyn..

[9] Dong J., Sun Z., Zhang P., Zhang J., Chen C., Qian R. (2026). Research on Autonomous Ship Route Planning Based on Time-Dynamic Theta* Algorithm Under Complex and Extreme Sea Conditions. Appl. Sci..

[10] Hu L., Zeng W., Chen C. (2024). Path planning for inspection robots based on improved D*Lite-APF algorithm. Mod. Electron. Technol..

[11] Liu G., Ma Y., Qi F., Xu Y. (2022). Urban Logistics UAV Path Planning Based on the Improved A*-Artificial Potential Field Method. Flight Dyn..

[12] Sun S., Sun T. (2022). Research on UAV Path Planning Based on the Fused A* Algorithm. Electron. Meas. Technol..

[13] Wu X., Guo M., Hu A., Wu Q. (2025). UAV path planning based on improved genetic particle swarm optimization algorithm. Chin. J. Sci. Instrum..

[14] He H. (2026). Research on UAV path planning based on improved ant colony algorithm. Intell. IoT Technol..

[15] Zhang H., Li H., Liu H., Xu W., Zou Y. (2020). Path Planning for Urban Logistics UAVs. Transp. Syst. Eng. Inf..

[16] Xie H., Han S., Yin J., Ji X., Yang Y. (2024). Safety Risk Assessment and 3D Path Planning for Low-Altitude UAVs in Complex Urban Environments. J. Saf. Environ..

[17] Li Y., Zhao R. (2024). Research on Multi-Objective UAV Path Planning in Complex Urban Environments. J. Nanjing Univ. Aeronaut. Astronaut..

[18] Fan J., Lei T., Han W., Wang R. (2021). A Review of UAV Trajectory Planning Techniques. J. Zhengzhou Univ..

[19] Zhang R., Wang W., Tian Z., Zhang W. (2022). 3D Flight Path Planning for UAVs Based on the Model-Constrained A* Algorithm. Int. J. Electron. Meas. Technol..

[20] Yue F., Wu Y., Li X., Dang Y., Zhang S., Wang L. (2026). Adaptive RRT* path planning for mobile robots integrating Bezier curves and sparse sampling. J. Beijing Univ. Aeronaut. Astronaut..

